# *microTrait*: A Toolset for a Trait-Based Representation of Microbial Genomes

**DOI:** 10.3389/fbinf.2022.918853

**Published:** 2022-07-22

**Authors:** Ulas Karaoz, Eoin L. Brodie

**Affiliations:** ^1^ Earth and Environmental Sciences, Lawrence Berkeley National Laboratory, Berkeley, CA, United States; ^2^ Department of Environmental Science, Policy and Management, University of California, Berkeley, CA, United States

**Keywords:** functional traits, functional guilds, ecological strategy, trait-based model, profile hidden markov model, microbial genome, fitness traits, trait inference workflow

## Abstract

Remote sensing approaches have revolutionized the study of macroorganisms, allowing theories of population and community ecology to be tested across increasingly larger scales without much compromise in resolution of biological complexity. In microbial ecology, our remote window into the ecology of microorganisms is through the lens of genome sequencing. For microbial organisms, recent evidence from genomes recovered from metagenomic samples corroborate a highly complex view of their metabolic diversity and other associated traits which map into high physiological complexity. Regardless, during the first decades of this *omics* era, microbial ecological research has primarily focused on taxa and functional genes as ecological units, favoring breadth of coverage over resolution of biological complexity manifested as physiological diversity. Recently, the rate at which provisional draft genomes are generated has increased substantially, giving new insights into ecological processes and interactions. From a genotype perspective, the wide availability of genome-centric data requires new data synthesis approaches that place organismal genomes center stage in the study of environmental roles and functional performance. Extraction of ecologically relevant traits from microbial genomes will be essential to the future of microbial ecological research. Here, we present *microTrait*, a computational pipeline that infers and distills ecologically relevant traits from microbial genome sequences. *microTrait* maps a genome sequence into a trait space, including discrete and continuous traits, as well as simple and composite. Traits are inferred from genes and pathways representing energetic, resource acquisition, and stress tolerance mechanisms, while genome-wide signatures are used to infer composite, or life history, traits of microorganisms. This approach is extensible to any microbial habitat, although we provide initial examples of this approach with reference to soil microbiomes.

## Importance

The rapid adoption of high-throughput microbial sequencing is leading to accumulation of microbial genomes at an ever-increasing rate. These genomes represent instances from not only isolated microbes but also microbial populations in their native environmental context as metagenome-assembled genomes (MAGs) or single-cell amplified genomes (SAGs). We believe that an ability to efficiently predict ecological traits directly from primary sequence data is a necessary interface between microbial *omics* information and trait-based microbial ecology, and success here will significantly advance our ability to uncover generalizable features of microbiomes and their environmental context. To streamline the process of going from genome sequences to putative ecological traits, we developed *microTrait,* a set of tools to efficiently discover and distill the trait-based representation of a microbial genome.

## Introduction

Linking microbiome structure and dynamics to ecosystem functioning globally in a predictive way and in face of global change has been a long-standing goal of microbial ecology (Finlay et al., 1997; Prosser et al., 2007; Van Der Heijden et al., 2008; Todd-Brown et al., 2012; Bier, Bernhardt et al., 2015). Efforts towards this goal traditionally included taxon-centric measurement approaches (Thompson et al., 2017; Ramirez et al., 2018) (Madin et al., 2020). Genetic, physiological, and ecological characterization of cultured isolates provided links between specific taxa and ecosystem processes like contributions to elemental and nutrient cycles, and biomass production. With the commoditization of high-throughput sequencing of taxonomic marker sequences, much effort in taxon-centric approaches shifted to extrapolating what is learned from representative isolates in the lab to their phylogenetic nearest neighbors detected with environmental community sequencing (Langille et al., 2013; Asshauer et al., 2015). Such approaches to infer functional groups via phylogenetic markers inherently assume strong phylogenetic conservation of microbial traits. Furthermore, without any whole-genome data, they are limited to taxa with cultured isolates.

Microbial-biogeochemical models are crucial tools in linking microbiome dynamics, environmental responses, and ecosystem processes across scales. Wide-spread availability of taxon-centric microbial measurements have naturally popularized taxon-centric models including few species or functional groups dominant at the local scale of interest. The upward scalability of such models would be limited given the fact that no single taxa would dominate at larger scales and with a limited number of parameter sets, the model would have poor adaptive capability both across scales and environmental conditions. Moreover, trying to approach the complexity of real systems at larger scales by adding more taxa or functional groups lead to increasingly complex models with a continuous demand for more parameters. Given these limitations of taxon-centric approaches in modeling the diversity and activity of microbes globally and with changing environmental conditions, trait-based representation of microbes is becoming increasingly popular.

Trait-based approaches represent an intermediate approach to modeling complex populations while also preserving key mechanistic properties that determine fitness in dynamic systems. The trait-based framework represents microbes with traits that can be summarized by few parameters and that are constrained by environmentally-dependent trade-offs. These approaches were developed in the field of plant ecology (Westoby and Wright 2006; Ackerly and Cornwell 2007), and have more recently been applied within microbial ecology at various scales, including global oceans and terrestrial environments (Follows et al., 2007; Allison 2012; Bouskill et al., 2012). The main underlying assumption is that combination of traits determines physiological performance which influences individual fitness and life history evolution. By abandoning the taxon concept, the trait-based framework strives to achieve a succinct description of the microbial communities with few essential communities, avoiding the complexity trap of taxon-centric modeling approaches. The challenge with this approach is to identify the key properties or traits of members of microbial communities and how these traits are regulated or trade-off against other traits, and to use this information to parameterize or constrain the functional potential of the modeled communities.

Traits may be identified through *‘omic* approaches (e.g. potential to produce or the detected activity of an extracellular enzyme, the genes for a specific metabolic pathway, the genomic capacity to replicate rapidly etc) or through physiological studies (e.g. enzyme, substrate uptake or growth kinetics, cell surface area, biomass stoichiometry, composition of storage pools etc.) or they may be inferred by manipulation experiments such as stable-isotope tracing with substrates at various concentrations to determine relative affinities. The paradigm shift from a taxa-to a trait-centric representation of microbiomes is partly stimulated by the wide-use of *omic* technologies to illuminate the functional potential of environmental microbial communities and their interactions with each other, higher organisms, and their environment (Sharon and Banfield 2013; Anantharaman et al., 2016; Gupta et al., 2016; Sangwan et al., 2016; Woodcroft et al., 2018). In particular, focusing on genome rather genes as ecological units makes the incorporation of many concepts from ecological and evolutionary theory into models possible therefore increase the value of the *omic* data for trait-based modeling (Prosser 2015). The rate at which isolate genomes, single-cell assembled genomes (SAGs) and metagenome-assembled genomes (MAGs) are being generated provide an unprecedented resource to study patterns in fitness trait conservation, trait linkage (i.e. co-occurrence patterns of traits within ecological units), trait trade-offs, and trait-environment relationships across scales. This continuous stream of microbial genomes necessitates development of computational tools that can efficiently and robustly extract potential traits from genome sequences.

Currently, the methods used to infer functional traits from genome sequences include 1) pairwise sequence alignments and database search (Shaffer et al., 2020), 2) statistical learning methods (Feldbauer et al., 2015; Weimann et al., 2016), and 3) phylogenetic inference (Goberna and Verdu 2016). Homologous inference from sequence alignments with tools like BLAST (Altschul et al., 1990), USearch (Edgar 2010), or DIAMOND (Buchfink et al., 2015) have large memory requirements and long run times, which makes these methods challenging to scale for a typical user to thousands of genome sequences. In addition, for the detection of remote homologs, the sensitivity of alignment-based methods is lower than the profile methods (Brenner et al., 1998). Statistical learning methods to predict microbial traits depend on the availability of extensive training sets to establish genotype-phenotype relationships. Such data exist only for a very limited set of core phenotypes and therefore the resulting models, while they can be highly accurate, offer a narrow view of the microbial trait space (Yabuuchi 2001; Ruan 2013). Phylogeny-based methods predict missing trait values of new genomes based on the traits of their evolutionary relatives. While phylogenetic conservatism of certain traits has been documented for bacteria and archaea, prokaryotic traits of ecological relevance have overall weak phylogenetic signal (Martiny et al., 2013). In addition, as the bulk of the current information on phenotypes are centered around organisms of biotechnological and medical interest, the accuracy of the phylogenetic trait prediction remains low (Goberna and Verdu 2016).

To fill this need, we developed an R package, *microTrait,* that provides a conceptual framework and associated pipelines to translate a microbial genome into a suite of potential fitness traits. *microTrait* maps a genome sequence into a hierarchical trait space that covers energetic, resource acquisition, stress tolerance, and life history traits that underlie microbial strategies describing environmental microbes (Malik et al., 2020). Our pipeline makes use of literature-supported *omics* markers defining trait-based microbial strategies to quantify trait profiles for microbial genomes. Given a genome sequence, individual gene markers are detected with a model-based approach using a new HMM database of protein families. The models have been trained with protein sequences that represent sequence diversity from genomes and metagenomes and their accuracy measured independently with KEGG orthology database. The traits are inferred from gene markers based on their presence/absence patterns and presented in a hierarchical manner.

## Results

### Microbial Traits With Genomic Basis

**FIGURE 1:**
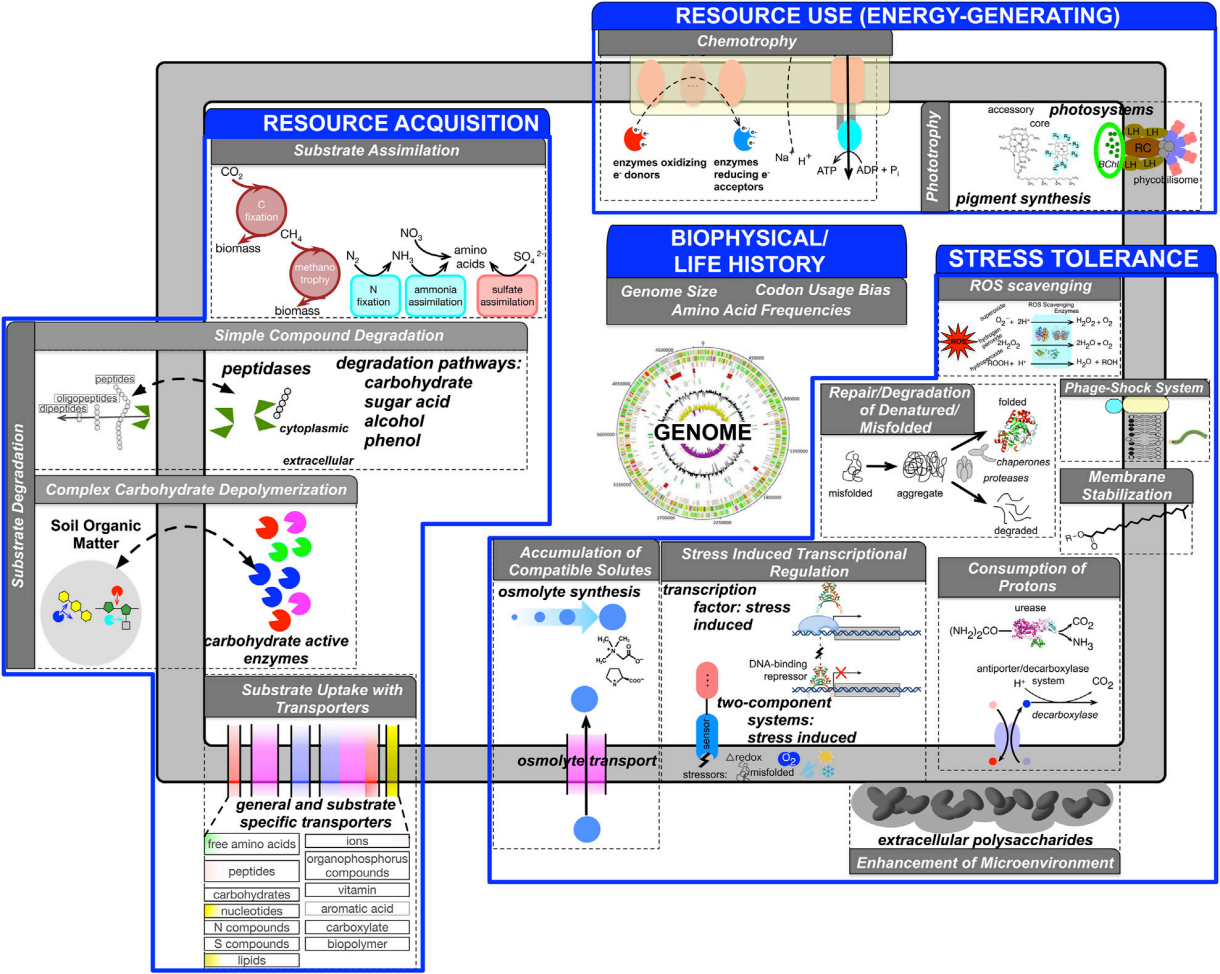
Conceptual overview of genome-derivable traits (gray boxes) underlying ecological strategies (blue boxes) represented in *microTrait* based on literature surveys. For each trait, genomic features are indicated. Supplementary Table S1 provides full details for the *microTrait* hierarchy. Supplementary Table S8 lists references for genomic features underlying ecological traits.

The overarching goal of our approach is to reduce the dimensionality and complexity of the genomic information such that a genome is represented as a feature vector where individual features represent one or more aspects of an ecological strategy (Lajoie and Kembel 2019). Microbial traits span a wide range of phenotypic, ecological, and metabolic characteristics. The choice of specific traits and their representational granularity depend on the research question of interest. We first review the genome based traits inferred by *microTrait,* rationalize their choice primarily following the frameworks proposed by (Green et al., 2008) and more recently (Malik et al., 2020) (Figure 1).

At the very fundamental level, our approach takes as input a genome sequence and maps it to a trait space in a computationally scalable way. Here we adopt a microbial counterpart of the widely used definition of “functional traits” for macroorganisms as measurable characteristics that “impact fitness of an organism via its effect on growth, reproduction, or survival” at the individual level (Violle et al., 2007; Violle et al., 2014). Unlike for macroorganisms, measuring traits at the individual microbe level in complex communities is currently not feasible, although single-cell imaging and ‘*omic* technologies are beginning to expand our understanding of population heterogeneity at these native scales (Wang and Bodovitz 2010; Bock et al., 2016). Genomes have recently been proposed as the ecological units (Prosser 2015; Turaev and Rattei 2016) at which genome-inferred traits should be measured. Advances in DNA sequencing and computational protocols has led to a more or less continuous stream of provisional genomes not only from cultured isolates but also from single-cells (SAGs) and metagenomes (MAGs) (Sharon and Banfield 2013). Though as an ecological unit, the resolution represented by MAGs may not currently match its counterpart for macroorganisms, possibly representing mosaics and distorting or masking intra-population differences, they nevertheless provide an unprecedented window into complex microbiomes and provide especially valuable insights into the physiology and metabolism of uncultivated organisms in their natural environments. As such, a genome-centric lens to traits allows scaling of organism level traits to communities (through incorporation of genome abundances) and therefore at larger scale as well as studying trait linkage across ecologically relevant units.

We identified genomic features that can be mapped to microbial ecological strategies, conceptualized under four dimensions (Figure 1) organized as a hierarchy (“*microTrait* hierarchy”: Supplementary Table S1). Within each strategy, the trait information is organized as a hierarchy whose leaf nodes map to specific genome derived features. Supplementary Table S8 lists the full list of references that establish the links between each genome derived feature and the ecological strategy at the most granular level. Here we give an overview of the traits for each ecological strategy:

### Resource Acquisition Traits

A tremendous variety of substrates ranging from simple inorganic ions to complex organic molecules serve as resources for microbes. Microbes have adapted a suite of concrete strategies with genomic basis to be competitive in a wide range of environments with spatiotemporally variable resource profiles. Many microorganisms have the potential to produce exoenzymes that can disassemble complex resources (substrate degradation), which can then be acquired through uptake (substrate uptake) via membrane transporters (Berntsson et al., 2010; Arnosti 2011; Zimmerman et al., 2013; Arnostil et al., 2014; Courty and Wipf 2016; Bergauer et al., 2018). Thus, one aspect of resource acquisition strategy concerns the investment in both the number and diversity of exoenzymes and membrane transporters a microbe would maintain in a microbial genome. Substrate uptake is linked to substrate assimilation traits that determine the capacity for assimilation of inorganic compounds.

### Resource Use (Energy Generating) Traits

Redox reactions underlie all biological energy metabolism and redox chemistry provides an organizing principle to connect microscale to global scale processes (Falkowski et al., 2008; Ramirez-Flandes et al., 2019). Genes whose protein products catalyze redox reactions, their coupling to energy conservation, and their genomic organization determine the basis for microbial metabolic strategies. Historically, in the pre-genomic era, single metabolic traits were evaluated in isolation to define “metabolic functional groups” but genomic data has underlined the tremendous metabolic flexibility of microbes (Anantharaman et al., 2016). As a result, classical enumerations of microbial metabolism are not sufficient to represent the linkage of metabolic traits. Representation of microbes as a suite of energy metabolism traits provides a more complete picture and a data driven definition of metabolic guilds.

### Stress Tolerance Traits

Stress may be induced by physical, chemical, or biological conditions that adversely affect microbial growth and survival. Microbes that use stress tolerance strategies respond to a variety of stressors using several physiological and evolutionary mechanisms. Though the specific stress response depends on the particular suboptimal conditions, common traits with genomic underpinnings have been broadly identified (General Stress Tolerance Traits). These include increasing the concentration of some molecular chaperones (stress proteins/heat-shock proteins) to combat biomolecular damage in response to stress. This is a universal feature across all domains of life but the relative importance of genetic (i.e., diversity and gene copy number) or regulatory (transcriptional, translational, and post-translational) processes under different stressors is less clear (Feder and Hofmann 1999; Hecker and Volker 2001; Yu et al., 2015).

Genomic bases of microbial traits that underlie stress tolerance to specific physiochemical and chemical factors have also been identified: 1) Temperature stress: a suite of heat shock genes serving as chaperones and proteases are involved in the protection, repair, and degradation of denatured/misfolded proteins. Response to cold shock involves adaptation of the membrane via an increase in the proportion of unsaturated fatty acids and activation of chaperone cold shock proteins to restore mRNA functionality. 2) Desiccation, osmotic, salt stress: Known molecular strategies to tolerate drought and freezing include production or uptake of osmolytes like trehalose and glycine betaine to reduce water potential and maintain hydration or synthesis of extracellular polymeric substances (Csonka 1989; Ko et al., 1994; Mindock et al., 2001; Costa et al., 2018). 3) Oxidative stress: The response to oxidative stress is a complex one that involves the coordinated regulation of many genes most critically involving enzymes that scavenge reactive oxygen species. The activation of such regulons requires redox sensing (two-component redox sensors and redox-sensitive TFs). 4) pH stress: Similarly to general, oxidative, and temperature stress, molecular mechanisms for protection from acid stress include investment in chaperones, proteases and the ability to sense and respond to redox conditions through two-component systems and TFs. Unique mechanisms for maintenance of intracellular pH include the consumption and extrusion of intracellular protons by acid-inducible amino acid decarboxylase-antiporter and urease systems, and the enzymatic conversion of unsaturated fatty acids into cyclopropane fatty acids.

### Life History Traits

Ecological and evolutionary processes leave their signatures in overall microbial genome content and organization. A key dimension of any ecological strategy is growth. Optimal growth characteristics of microbes are key to understand how the key traits regarding resource acquisition, resource use, and stress tolerance are realized to adapt to a particular environmental niche. Traits that concern these characteristics are classified as life history traits. Codon usage bias and ribosomal RNA (rRNA) operon copy number are linked to maximum growth rate, a life history trait constraining all other functional traits (Weider et al., 2005; Vieira-Silva and Rocha 2010; Weissman et al., 2021). Another key life history trait closely linked to the overall genomic adaptation is optimal growth temperature (OGT). Temperature is a master regulator of enzyme activity and overall cell machinery. A combination of quantifiable proteome-wide features predictable from genome sequences allows OGT to be hypothesized solely from genomic sequence (Zeldovich et al., 2007; Sauer and Wang 2019).

### *microTrait* Pipeline

**FIGURE 2:**
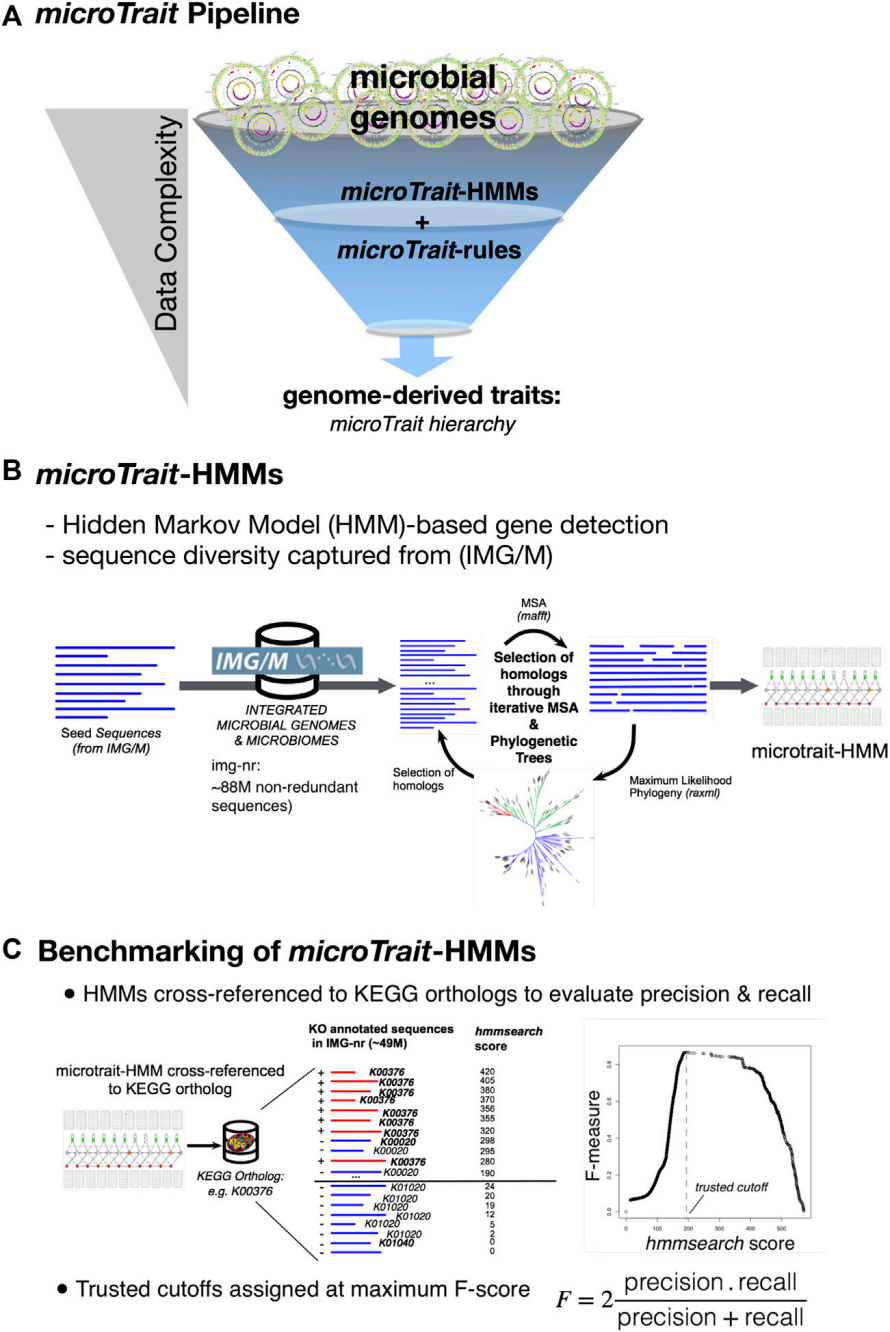
Overview of *microTrait*. **(A)** *microTrait* pipeline consists of a library of gene-level Hidden Markov Models (*microTrait*-HMMs) for detection of genome features and logical rules (*microTrait*-rules) that map these features to traits. The output from the pipeline are trait matrices (genomes × traits) at different granularities corresponding the levels of the *microTrait* hierarchy. **(B)** Workflow for construction of *microTrait*-HMMs. Each HMM models the diversity of sequences from IMG/M at gene-level. **(C)** Benchmarking of *microTrait*-HMMs. The trusted cutoffs for *microTrait*-HMMs were determined through cross-references to KEGG orthologs (whenever available).

The computational pipeline to infer traits from primary genome sequences has two major components (Figure 2A): 1) a database of gene HMMs (*microTrait-HMM*) to model the diversity of protein families based on sequences from genomes and metagenomes with independently established accuracy to detect genetic loci (Figure 2B and Supplementary Table S2), 2) a set of rules (*microTrait* rules) encoded in predicate logic to infer traits from presence and absence of the set of loci modeled in *microTrait-HMM* (Supplementary Table S4)*.* The model-based detection of genetic loci ensures decreased run-times and interoperability across datasets (given model and scoring cutoff). The rule-based framework to infer traits from primary features gives the user the flexibility for redefinition and refinement.

### Cross References to External Databases From *microTrait*-HMM

The statistical models in *microTrait-HMM* reflect the most recent sequence diversity from both cultured and uncultured microbes and therefore should have improved accuracy over existing methods to detect genes underlying traits covered in *microTrait*. To ensure interoperability of the *microTrait* pipeline with the existing HMM databases and relevant sequence database resources, for each gene model we provide database cross references to KEGG (Kanehisa and Goto 2000), Transporter Classification Database (Saier et al., 2016), and Enzyme nomenclature database (through EC numbers) (1999).

### Performance of Gene HMMs and Assignment of Trusted-Cutoffs

We assessed the performance of each *microTrait-HMM* by first determining the corresponding orthologous group (KO number) in KEGG orthologs database (when the loci was represented in KEGG) (Figure 2C). A test dataset for the gene model in question was built by using IMG/M sequences labeled with the determined KO number (“true positives”) and the remaining KO numbers (“true negative”). IMG/M database was scanned with the profile HMM using HMMER/hmmsearch. F-scores (harmonic mean of precision and recall) were calculated as a function of “hmmsearch scores” based on the test dataset with R using ROCR package (Sing et al., 2005). The smallest score that maximizes F-scores was assigned as the trusted cutoff. Supplementary Table S3 summarizes the performance of each model in *microTrait-*HMM. Overall, at the determined trusted cutoffs, the overwhelming majority of *microTrait-*HMMs (94.2%-1,686 out of 1790 HMMs) had high sensitivity (≥75%) and low FPR (false positive rate), with 92% of HMMs having an F-score >=0.8 (Supplementary Figure S1).

### *microTrait* Pipeline: Derivation of Traits From Genome Sequences

**FIGURE 3:**
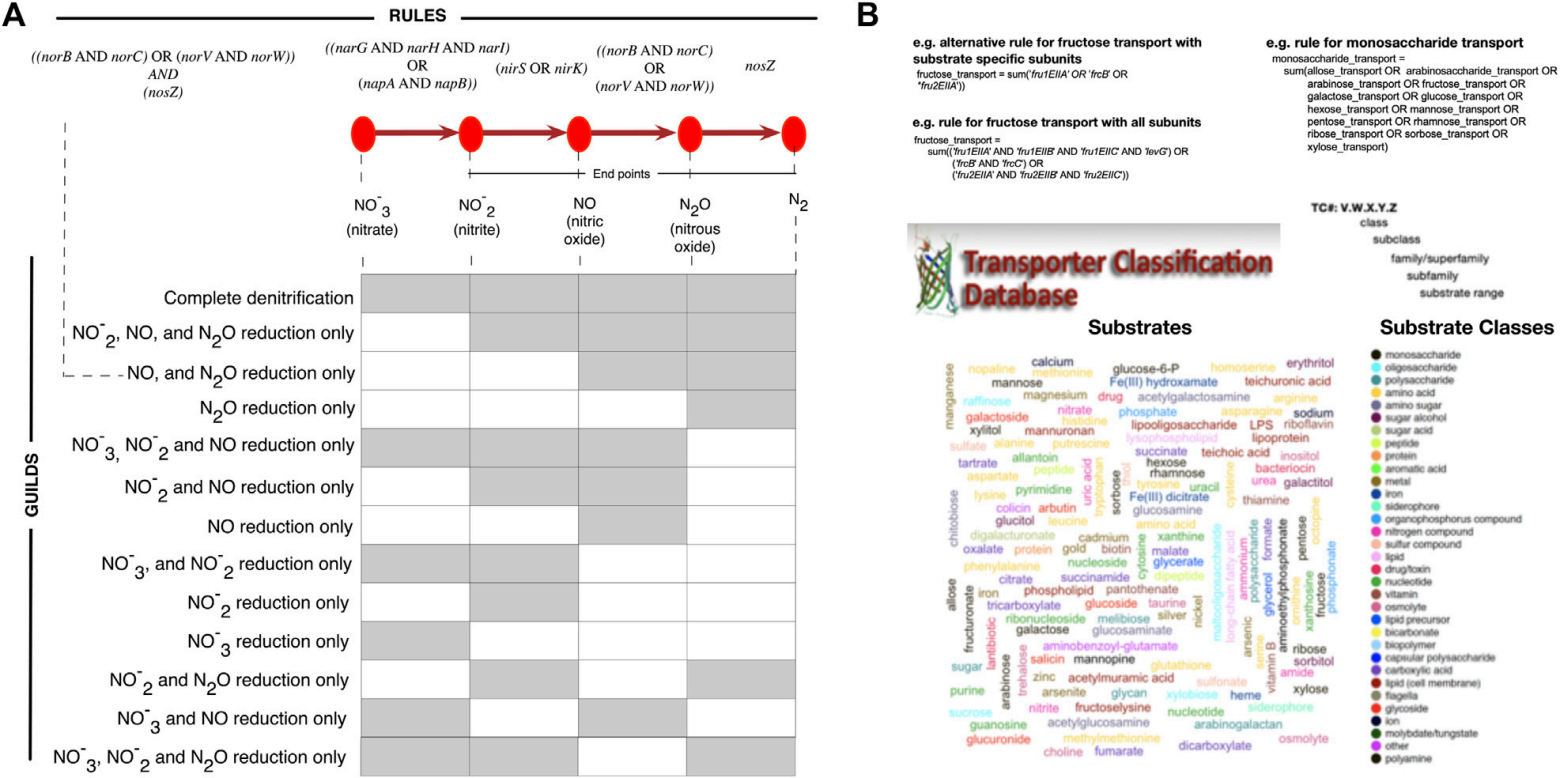
Trait inference with *microTrait* rules. microTrait rules use simple boolean logic to map presence/absence of *microTrait*-HMMs (italicized) to traits. The reconfigurability of the rules makes the exploration of the effect of different trait definitions on the microbial guilds possible and therefore enables a flexible microbial trait extraction pipeline. Examples for trait definitions from rules for **(A)** denitrification traits. Rule-based inference allows flexible definition of traits, for example by end products of denitrification. **(B)** substrate uptake. *microTrait* represents substrate uptake traits using the range substrates documented in TCDB (Transported Classification Database) (shown as word cloud colored by substrate class). Traits relevant to the uptake of substrates (example for monosaccharides) can be defined in a hierarchical manner with rules defined from other rules and *microTrait*-HMMs.

The input to *microTrait* is a genome sequence (.fa) or the corresponding protein coding genes (.faa) in FASTA format. When genomic rather than protein coding gene sequences are supplied, Prodigal is used to predict open reading frames (Hyatt et al., 2010). For each genome, protein sequences are scanned against *microTrait-HMM* with HMMER/hmmsearch to generate a count table for the detected gene models. Binary and continuous traits are assigned using the count table and predefined logical rules mapping the presence/absence of genes(s) or other rules to specific traits (Figure 3). The rules can be edited by the users within the R package. Their role is twofold: On one hand they allow modifications in the way some binary traits can be defined (for instance based on one or more proteins in a large complex, or one or more steps in a pathway) giving the user flexibility. They can also be used to increase detection sensitivity for provisional or lower quality genomes (i.e., SAGs and MAGs).

### Modular Trait Definitions With Predicate Logic

*microTrait* uses Boolean algebra to map protein family content into traits through *microTrait* rules (Supplementary Table S5). In this framework, each protein family is a Boolean variable (i.e. equals 1 if detected, 0 otherwise) whose value is determined by the output of the corresponding *microTrait-*HMM. The traits are represented by rules whose arguments are one or more protein families, other rules, or a combination of these. Conceptually, the rules map to representations of protein complexes with multiple subunits or a series of enzyme catalyzed reactions that transform one molecular species into another. While the standard package comes with a predefined set of rules, the rules themselves and the mapping of rules to traits are modular and can be modified by the user. As an example, consider denitrification traits (Figure 3A). The canonical denitrification pathways, excluding accessory and regulatory proteins, involve 4 protein complexes (NarGHI: the inner membrane-bound nitrate reductase; NapAB: the periplasmic nitrate reductase; NorBC, NorVW: nitric oxide reductases) and 3 proteins (NirS, NirK: nitrite reductases; NosZ: nitrous oxide reductase). Together, these are represented by 12 protein families (italicized gene names in Figure 3A) and the four individual enzymatic steps are represented by 4 rules. From these rules, several denitrification traits corresponding to individual functional guilds can be defined.

For transporters and polymer specific extracellular enzymes, we compiled a list of the experimentally reported substrates of each enzyme using the Transporter Classification Database (TCDB) (Saier et al., 2016) and the Database of carbohydrate-active enzymes (dbCAN) (Yin et al., 2012). We then classified each reported substrate into broad substrate classes (Figure 3B and Supplementary Table S6). The relevant rules for transporters and extracellular enzymes let the user quantify the number of protein complexes with a given substrate or substrate class.

A challenge in assigning traits to genomes based on the protein family signatures is the modularity of the underlying pathways. This modularity might be truly reflecting the genomic variation within a set of isolates, MAGs or SAGs but also be an apparent manifestation of incomplete and noisy genomic information. Starting with genomic sequences, *microTrait* allows the investigation of this modularity across a set of genomes. The resulting information can be used by the user to define custom logical rules to assign traits based on the protein family content.

### Comparing *microTrait* With a Taxonomy-Based Inference of Microbial Functional Groups

Linking taxonomic classification with function is a commonly used method to infer microbial traits. Faprotax is a manually curated database that maps taxa to functional groups based on the physiological studies for the cultured representatives of these taxa (Louca et al., 2016). The taxonomic resolution is typically at species or genus level but can also be less specific (i.e. family or higher). Using a large collection of isolate genomes from environmental ecosystems (refer to Materials and Methods for construction of the genome collection) and literature references for functional affiliations based on taxonomic names in Faprotax (Supplementary Table S11), we have quantified the extent to which *microTrait-*rules recovered the validated culturable taxa for different microbial functional groups. For each functional group, we first matched the taxonomic names from literature, primarily genus/species names but also extending to higher ranks for certain functional groups, to canonical NCBI taxonomic names. All available genomes from environmental ecosystems with the respective taxonomic affiliation were considered as a “positive” for that functional group according to the Faprotax approach (Supplementary Table S12). We have then tested how many of these assumed Faprotax positives the *microTrait* pipeline was able to recall solely based on the functional trait predictions from genomes. In addition, for each functional group, we have also evaluated the specificity of genome-based calls based on the assumption that all negatives via the Faprotax taxonomic affiliation were “true negatives” (Supplementary Table S13).

Among 41 functional groups, 29 had a recall rate over 70%. Functional groups for which *microTrait* had low recall rates included anammox (0 *microTrait*+ genomes out of 7 Faprotax+ genomes; 0/7), dark iron oxidation (10/16), iron respiration (19/86), aerobic nitrite oxidation (6/13), chlorate reducers (3/6), dark sulfide oxidation (49/93), anoxygenic photoautotrophy Fe oxidizing (9/16), dark sulfur oxidation (71/124), sulfur respiration (82/139), thiosulfate respiration (88/145). A close examination of the taxonomic identity of the genomes “missed” by *microTrait* suggested a variety of explanations for the functional groups with poor recall.

A primary advantage of inferring microbial traits directly from genomic sequences rather than by taxonomic names is the ability to resolve diversity (species or strain level), which increases the prediction accuracy. We have observed that for many functional groups defined in Faprotax, the genomes that were assigned to the taxonomic clades lacked the required genetic repertoire for the metabolic function in question. Some prominent examples are for the “anammox” and “dark iron oxidation”. For anammox, among the diversity of taxa (genus and species), only *P. mendocina* had corresponding genomes in the isolate set (n = 7) and none of those had the genomic features for anammox suggesting that this is a strain specific trait for *P. mendocina.* Similarly, for dark iron oxidation, genome features suggested that the trait can be strain specific. Among 15 *R. palustris* and 2 *M. ferrooxydans,* a limited number (9 and 1 genome respectively) was genome-supported to carry the trait. There were also cases where the genomic evidence suggested that trait conservation was limited to deep taxonomic levels so a taxonomic inference at genus or family level would have impacted the accuracy of Faprotax method. For instance, methanotrophy is associated with Methylocystaceae (family) and Methylocapsa (genus) yet the trait was specific to subfamily/subgenus. Among 7 Methylocystaceae genera with genome representatives, 2 genera (Methylocystis and Methylosinus) had genome support for the trait. Similarly, 2 out 3 Methylocapsa species with genomes had evidence for the trait.

It should be noted that, there were also cases for which the absence of the genomic signal reflected limited knowledge for the genetic underpinnings of the trait. A typical example was for iron respiration, a trait for which current evidence suggests that electron transport for iron reduction proceeds in a different and unknown mechanism in acidophiles compared with *Ferrimonas* and *Shewanella* (Malik et al. 2018). Another example was for chlorate reduction, a process whose genomic trait sits in a region prone to horizontal transfer (Clark et al., 2013) which impacts the accuracy of a gene-level profile HMM approach. Overall, these disagreements between taxonomic and genome-based approaches suggests that, a genomic feature-based approach such as *microTrait* increases prediction accuracy and precision, even when one considers single traits (such as functional groups).

### High-Throughput Extraction of Microbial Traits from Genomes with *microTrait*

As an example of scalable extraction of traits from genomes, we applied *microTrait* to publicly available isolate genomes and MAGs. The datasets we used included 1) isolate genomes from environmental ecosystems from IMG/M (n = 6,157), 2) MAGs from an aquifer system (n = 2,545) (Anantharaman et al., 2016), 3) MAGs from a thawing permafrost (n = 1,530) (Woodcroft et al., 2018), 4) MAGs from hydrothermal sediments (n = 666) (Dombrowski et al., 2018), and 5) MAGs from publicly available metagenome samples, referred to as Uncultivated Bacteria and Archaea Dataset (UBA) (n = 7,902) (Parks et al., 2017). This compendium of datasets (genome compendium) resulted in a total number of 20,062 genomes.

We tested *microTrait* on a machine with a 2.3 GHz 16-core Intel Xeon Processor E5-2,698. When run using a single core, with a single genome processed using that core, *microTrait* processed that genome in 3.94 ± 2.59 min, with an average of 1.11 min/Mb of genome sequence (Supplementary Figure S2). From these, we predict that *microTrait* can process an average microbial genome of size 4 Mb in approximately 4.5 min. In all runs, the memory footprint of *microTrait* was not larger than 60 MB. In a multiprocessor compute environment, *microTrait* is easily parallelizable using a typical data-level parallelization scheme (for instance using R’s *parallel* package (distributed as part of R-core)) mapping genomes to separate logical processors. In our tests, when run in a 64 processor compute node, the processing of the compendium of 20,062 genomes (total size = 47.9 Gb) took 12.47 h.

### *microTrait* Trait Matrix

When applied to multiple genomes, *microTrait* outputs a trait matrix of “genomes x traits” with three types of qualitative variables. Binary trait variables are calculated as presence/absence of a specific functional capacity and span 1) energy generation via specific electron acceptors/donors, 2) capacity to degrade, assimilate, or acquire specific substrates. Continuous trait variables are of two groups. The first group of continuous traits are calculated starting from counts of specific functional capacities in the genome and span 1) acquisition of chemical classes of substrates with transporters or via extracellular breakdown, 2) investment in extracellular polysaccharides and osmolytes. For each genome, the counts are normalized by genome size. The second group represent life history traits and include 1) minimum generation time (unit: h^−1^) predicted based on indices of codon-usage bias in ribosomal protein genes (a proxy for highly expressed genes) (Vieira-Silva and Rocha 2010) (Weissman et al., 2021), 2) optimal growth temperature (unit: °C) predicted from a suite of features derived from the nucleotide and protein sequences of the genome (Sauer and Wang 2019).

### Refinement of Functional Guilds Using *microTrait*

To exemplify the use of *microTrait* in refining functional guilds, we explored how denitrifier guilds can be defined based on the genomic distribution of denitrification traits in the isolate genomes from our compendium of genomes. Denitrification is a key biologically catalyzed process by which nitrogen available to plants is transformed to the atmospheric nitrogen pool as gaseous forms of nitrogen as molecular N_2_ or as an oxide of N. Denitrification occurs as a step-wise reduction of nitrogen oxides with gaseous products. Four reductases are involved in the denitrification, NAR, NIR, NOR and N2OR, sequentially catalyzing the reductions of NO3 - → NO2 - → NO →N2O →N2. Several previous studies reported both genomic and phenotypic evidence for truncated versions of the denitrification pathway but a global genomic analysis is not currently available (Sanford et al., 2012; Jones et al., 2014; Lycus et al., 2017; Liu et al., 2018; Gao et al., 2019).

We used the *microTrait* pipeline to explore all of the publicly available environmental genomes from the IMG/M database (Supplementary Table S9). This resulted in a “genomes X rules” matrix specifying for each genome whether each of the rules was asserted as TRUE or FALSE. The matrix was subset to rules underlying denitrification traits and the genomes were clustered based on their denitrification trait profiles. The clustering gave 13 denitrification-associated functional guilds, with 58.3% of the screened genomes involved in at least one denitrification-related process (Supplementary Figure S3). Only, a small proportion of these had the genomic capacity to perform complete denitrification to N2. Overall, the guilds correspond to generation of the same end products from different starting nitrogen compounds (e.g. guilds 1–4, 5−7, and 8−9 generating N_2_, N_2_O, and NO respectively), or multiple end products with missing steps (e.g. guilds 11–13). The default trait matrix in *microTrait* defines denitrification traits by the end products of denitrification (Supplementary Table S7) yet the workflow of going from genomic features to traits via *microTrait* rules makes redefinition of traits possible.

### Testing Trait Dimensionality of Microbial Genomes from a Given Ecosystem

*microTrait* hierarchy maps a microbial genome to a high-dimensional space of putative functional traits of ecological relevance. In trait-based ecological modeling, trait selection is of central importance not only for biological but also for computational, statistical, and practical reasons (Lajoie and Kembel 2019). In our conceptualization of the relevant traits for terrestrial ecosystems, the set of selected traits are assumed to approximate the intrinsic (i.e. true underlying but unobserved) dimensionality of microbial traits. Unlike for plants for which accumulated evidence suggests that the intrinsic dimensionality of functional trait space is low (Laughlin 2014), the intrinsic dimensionality of the trait space of microbes in specific ecosystems remains largely unknown. However, we can assume that if the selected trait proxies are largely independent of each other then, taken jointly, they should represent the underlying functional differences, and improve our ability to explain and predict microbial distributions.

**FIGURE 4:**
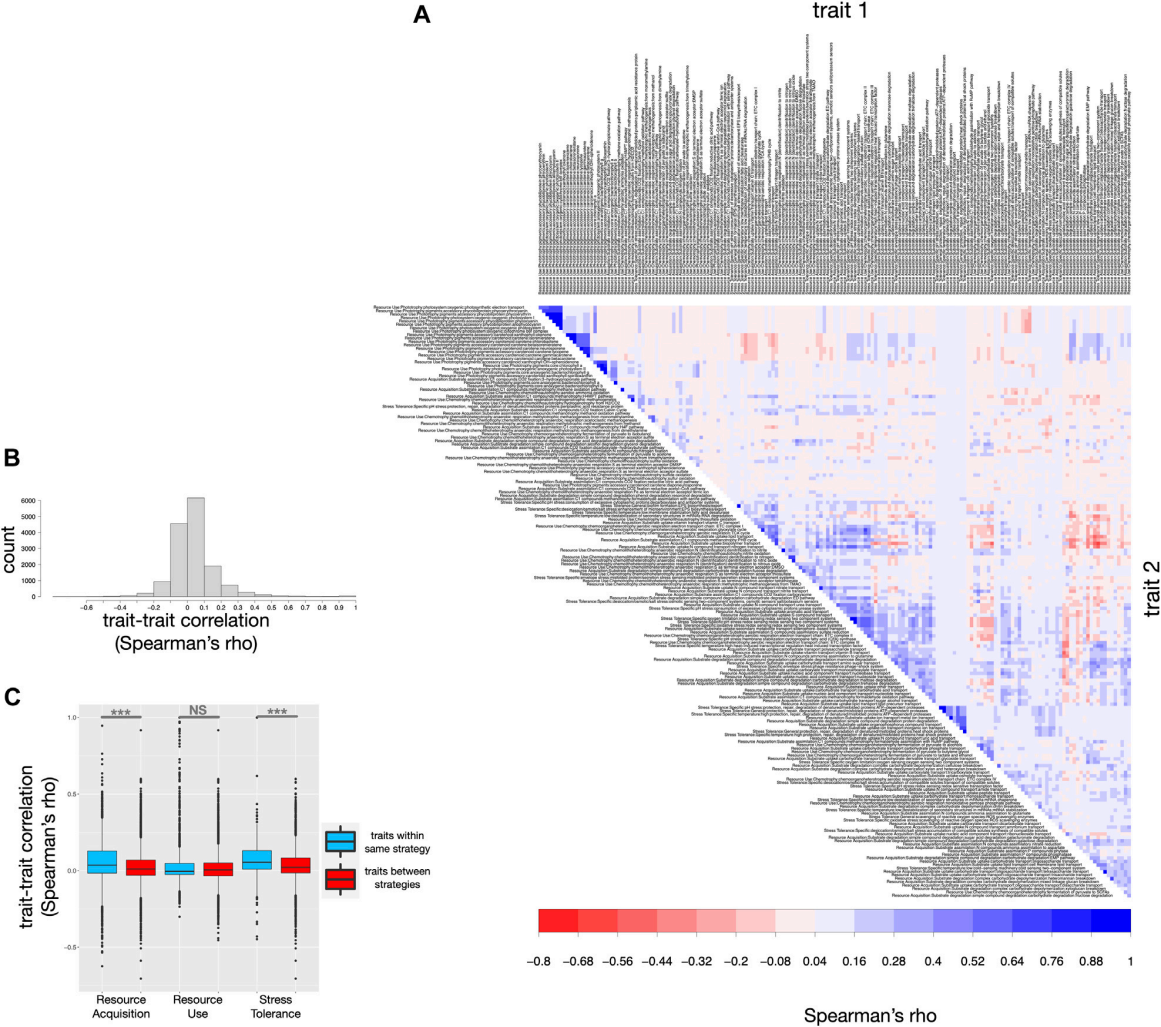
Correlation matrix for *microTrait* defined traits. The strength of the correlation (Spearman’s rho) is represented by the color intensity (positive: blue, negative: red). Left upper panel: the distribution of trait-to-trait correlation values, left lower panel: comparison of the distribution of trait-to-trait correlations within and between ecological strategies.

To investigate whether the selected traits in *microTrait* are largely independent, we used an extensive dataset of genomes of microbes isolated from terrestrial ecosystems to study the correlation structure of their *microTrait* profiles. The trait matrix (at granularity 3) for a total of 4,116 genomes of organisms isolated from terrestrial environments (ST9) was computed using *microTrait.* A non-parametric rank-order correlation metric was used to estimate the degree of relatedness between all trait pairs, visualized as a correlation matrix and reordered to elucidate the potential hidden structure and pattern in the matrix (Figure 4A).

Overall, the bulk of the correlations were weak (|ρ| < 0.3) suggesting that *microTrait* trait dimensions map to largely independent traits (Figure 4B). On the extremes, strong positive correlations would be indicative of redundancy of trait dimensions while negative correlations would be indicative of underlying tradeoffs for the ecosystem in question. Few strongly positively correlated blocks corresponded to phototrophic resource use traits linking the variety of phototrophic pigments and photosystems.

### Dimensionality Reduction with Guild-Centric Analysis of Microbial Genomes With *microTrait*

Metagenomics allow the recovery of the genomes of all detectable members of an ecosystem along extensive spatiotemporal gradients. The genomes then provide support for co-occurrence of ecologically relevant traits of the members that together underlie the ecosystem function. A typical genome-centric microbiome study involves the analysis of hundreds to thousands of genomes leading to trait matrices of high genomic dimensionality. This high dimensionality poses a particular problem for statistical analyses (Johnstone and Titterington 2009). Further, when attempting to leverage the information from these genomes for downstream modeling applications, there is both a practical need and discovery opportunities in quantify and reducing this dimensionality in a tractable manner. Organizing microbial members of an ecosystem community into “putative guilds” can reduce the dimensionality of a metagenomic dataset and hypothesize the functional niche of community members and computationally explore their interactions independently of their taxonomic origin. Here, using the soil ecosystem as an example, we show how to define microbial guilds in a data-driven manner using *microTrait.*

**FIGURE 5:**
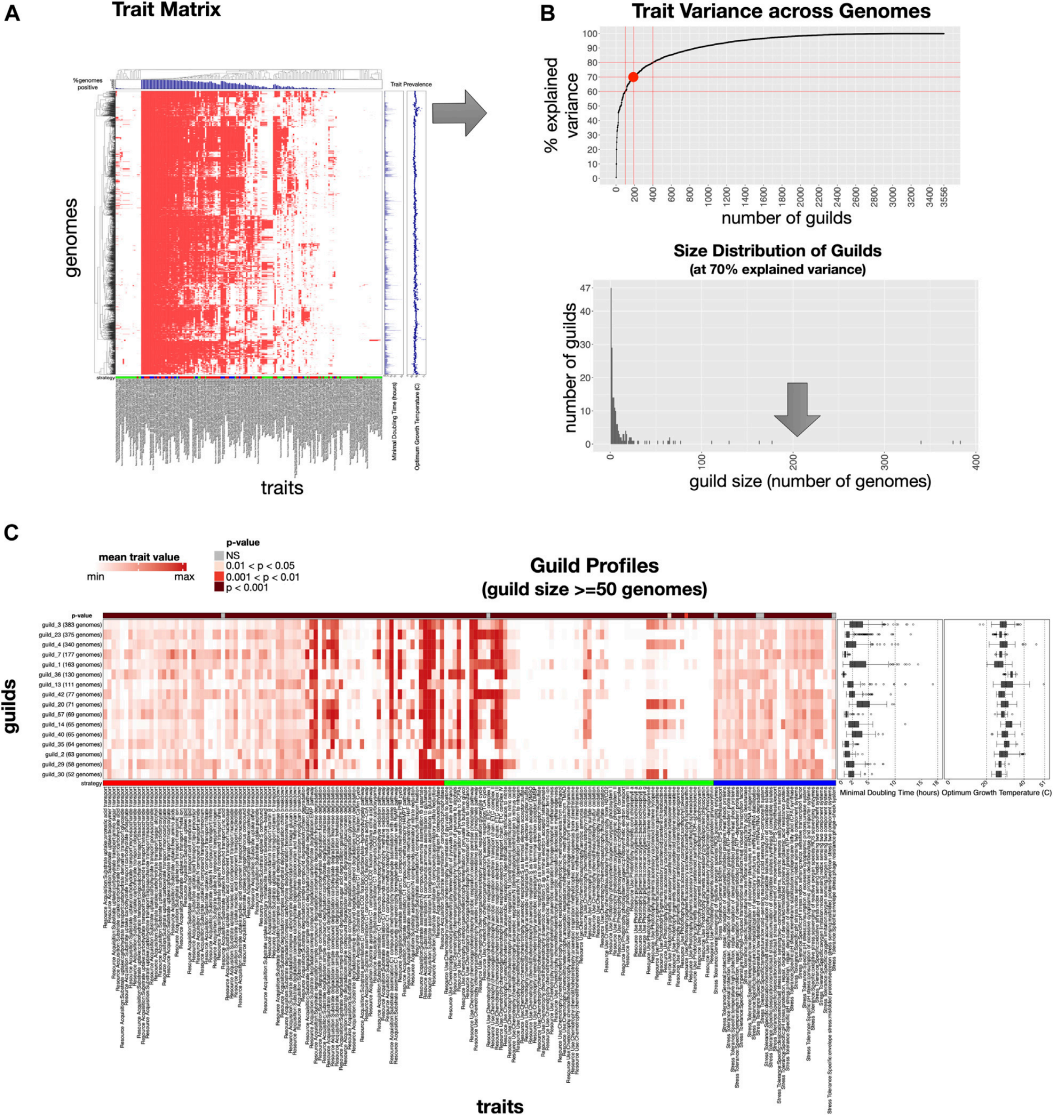
Primary use cases and graphical outputs of *microTrait* workflow. **(A)** Trait matrix provides clustering of a set of input genomes using trait profiles from *microTrait* outputs based on a distance metric taking into account mixed data types (i.e. for binary and count traits). Heatmap visualization use presence (red)/absence (white) of traits, with trait prevalence (% genomes positive) shown at the top panel. Life history traits (minimal doubling time and optimum growth temperature) are overlaid on the right panel in continuous scale. **(B)** Trait variance across genomes based on the genome clustering is quantified as a function of the number of guilds using analysis of variance using distance matrices. Guilds can be defined either at a fixed number of guilds or based on percent explained within-guild variance, which results in a size (number of supporting genomes) distribution of guilds. **(C)** Visualization of trait profiles for the defined guilds (guilds × traits), with mean trait values visualized across a color scale. Traits are ordered by ecological strategies (red: resource acquisition, green: resource use, blue: stress tolerance). For each trait, top panel shows the statistical significance of comparison of mean trait values across guilds. The distribution of life history traits are shown on the right side panels.

Given a set of genomes representing a habitat, *microTrait* can be used to discover and define functional guilds, parameterize the defined guilds with life history traits (minimum doubling time and optimal growth temperature), and reduce the dimensionality of the trait space in a quantifiable way. Figure 5 outlines the guild-centric pipeline starting with a trait matrix leading to the definition and characterization of the microbial guilds. Since *microTrait* encompasses both continuous and binary traits, the similarity between genomes are measured using a distance metric suitable for mixed data types (Wishart 2003) (see Methods). The resulting distance matrix (genomes x genomes) is clustered with unsupervised hierarchical clustering, visualized with trait presence/absence (i.e., treating continuous traits as binary variables), and annotated with the distribution of life history traits and trait prevalence across the dataset (Figure 5A). Quantifying relationships between genomes based on their trait profiles gives the opportunity to dynamically define guilds in a data-driven way for any dataset. The proportion of inter-guild variance explained can then be quantified as a function of the number of guilds (Figure 5B). A larger number of guilds corresponds to a smaller information loss at the expense of greater complexity for downstream applications. The user decides here where to operate along the curve depending on the shape (rate of change in steepness with increasing guilds) and the application of interest. Once determined, the guilds can be defined which results in a list of guilds, each representing a number of genomes and the joint distribution of traits captured by them. It is often useful to examine the distribution of the number of genomes that underlies each guild as on average the within-guild trait variance would be higher for guilds supported by a smaller number of genomes. The user can filter the guilds by number of genomes to generate a dataset that represents guild profiles, that is a fingerprint of the co-occurrence of traits for each guild and the within-guild distribution of life history traits (Figure 5C and ST 16).

We applied the *microTrait* data-driven guild-definition pipeline to soil isolate genomes from IMG (3,430 genomes with GOLD Ecosystem Type = “Soil OR Rhizoplane OR Rhizosphere OR Root”). All traits except “anaerobic ammonia oxidation (anammox)” were detected at least once in the dataset resulting in a trait matrix of dimensionality 3,430 genomes X 190 traits. To date no pure culture isolates of anammox organisms have been obtained (Jetten et al., 2005). Clustering analysis indicated that a total of 196 guilds captured 70% of the inter-guild variance, with 16 guilds supported by at least 50 genomes. Comparison of the trait profiles across guilds elucidates the differentiating trait features of a set of guilds with respect to other guilds.

For example, the top three guilds supported by the highest numbers of genomes (guild 3, guild 23, and guild 4; 383, 375, and 340 genomes respectively) were each enriched in specific traits under resource acquisition and resource use strategies (ST16). Guild 23 compared to guild 3, and 4 was marked by enrichment of the ability to assimilate simple C compounds, use 2 C compounds in the absence of glucose via glyoxylate cycle, uptake a variety of N compounds (elemental N and urea) as well aromatic acids and biopolymers, and fix elemental nitrogen for biomass. On the other hand, compared to guild 23, guild 3, and 4 represent a different strategy for incorporation of N compounds into biomass through assimilatory nitrate reduction and a unique ability to assimilate P compounds. Notably, although all three guilds were enriched in the capacity to utilize glucose, guilds 23 and guilds 3, and 4 differed in their preferred glycolytic pathways (canonical Embden-Meyerhoff-Parnass (EMP) pathway in guilds 3, and 4 vs. less common Entner–Doudoroff (ED) pathway in guild 23) reflecting differing preferences in balancing production of ATP (energy yield) and cost of protein synthesis to achieve maximum fitness (Flamholz et al., 2013). Across these three guilds (3, 23, and 4) differences in enrichment for stress tolerance mechanisms were not apparent, however, other guilds did display enrichment in specific stress tolerance strategies. For instance, among all the guilds supported by at least 50 genomes, guilds 7 and 14 were uniquely enriched in traits for desiccation and pH stress tolerance respectively.

## Discussion

Genome sequencing, from a data perspective, now provides a primary window into the traits that regulate fitness and function across Earth’s microbiomes. Genomes are increasingly recognized as a fundamental unit in the study of microorganisms, however, the integration of this information is required to understand how such genome units relate to ecologically coherent behavior. Exploration of feedbacks between microorganisms and their environments requires numerical modeling approaches, and the assimilation of genomic information has substantially lagged its generation. This assimilation of microbiome information into numerical models in an automated fashion remains a significant challenge as microbial communities are ultra-diverse, physiologically plastic, and dynamically adaptive. Trait-based approaches to microbial ecology provide a framework to represent microbial diversity in a way that facilitates prediction, integration and generalization (Lajoie and Kembel 2019) and the rate at which isolate and metagenome-assembled genomes are being generated provide an unprecedented resource to explore patterns in microbial trait conservation and linkage. The resulting information can be used to initialize and parameterize mechanistic trait-based models spanning a scale of complexities to explore the drivers of patterns in the distribution and co-occurrence of microbial traits. With *microTrait*, our goal was to provide an extendable toolset and computational pipeline to infer microbial traits from genomic data and show how the resulting information can be used to define microbial guilds with varying parameters.

Our approach to infer ecological traits from genomic data couples profile search methods with reconfigurable simple predicate logic. This coupling provides important advantages for deriving microbial traits from large numbers of phylogenetically diverse microbial genomes. Profile methods represent information across a family of evolutionarily related sequences from a multiple sequence alignment and increase sensitivity by incorporating position-specific information into a model. Moreover, the set of sequences from which gene-level *microTrait-HMMs* have been trained were selected from an extensive sequence database (IMG/M (Chen et al., 2019)) that not only includes genomes of cultured isolates but also MAGs and SAGs, the majority of which had been derived from environmental samples. Given that the bulk of the stream of incoming genomes from new studies is expected from MAGs with higher phylogenetic diversity compared to isolate genomes, the ability to detect remote homologs underlying microbial traits and explore sequence diversity from environmental samples is critical to increase the accuracy of trait prediction. With future releases of IMG, new sequences can be incorporated into multiple sequence alignments and consecutively *microTrait-HMM*s can be updated.

To benchmark and determine the score thresholds for each gene-level *microTrait-HMM*, we used the corresponding genes from the corresponding KO (KEGG Orthology) group. While this approach makes a systematic assessment of model accuracy possible by balancing model precision and recall, it should be noted that the computed thresholds may be overly strict for certain applications. Sequences in the KO database correspond to a highly curated set of sequences with a limited phylogenetic scope, this may lead to high precision and low recall with respect to the true labels especially for phylogenetically divergent or novel genomes not well represented in KEGG (Jaffe et al., 2020). Since the true orthologs for the underlying protein families are not known but can only be inferred, the accuracy of the model can only be estimated using independent labels such as those from KEGG. For applications where a higher recall at the expense of a lower precision is desired, it would be desirable to lower the HMM cutoff thresholds depending on the user input. We leave the implementation of such modifications for future work.

In this work, we focused on mechanistically well-studied traits whose genetic underpinnings have previously been documented and which can be conceptualized as Boolean rules. In addition to extraction of microbial traits with a rule-based system, further opportunities exist for unsupervised discovery of traits. For example, genomes with metadata labels determined experimentally or through text-mining (Alneberg et al., 2020) (Brbic et al., 2016) indicating the ecological niches of the organisms can be leveraged for exploring the genetic basis of organismal adaptation. Statistical modeling of the organismal niche and inference based on domain or gene content would be the classical approach towards this (Zhalnina et al., 2018; Ceja-Navarro et al., 2019). In addition, the exponential increase in the availability of high-quality MAGs with rich metadata will make feasible machine learning approaches that focus on prediction rather than explainability using a much larger number of features also feasible (Drouin et al., 2019).

Despite the increasing availability of genomic and physiological data of microbes, the adoption of trait-based approaches in microbial ecology is relatively recent. Unlike plants and animals, working definitions of microbial traits and conceptual frameworks to define functional guilds from these are lacking. The large diversity of microbial lifestyles manifest as a large number of potential traits some of which might be unobserved. Even with thousands of diverse genomes, the high-dimensionality of the potential trait space poses a challenge to define functional guilds for microbes. Here we adopted an operational definition of microbial guild as “groups consisting of diverse microorganisms with similar traits” based on a synthesis of a relatively small number of master traits that define microbial lifestyles. Depending on the specific analysis goals, a user might want to fine tune the granularity at which traits are defined (e.g., selection of different pathway endpoints as in denitrification or transporter/enzyme substrate classification). In *microTrait*, the reconfigurability of the rules makes the exploration of the effect of different trait definitions on the microbial guilds possible and therefore enables a flexible microbial trait extraction pipeline.

Finally, a trait-based microbial ecology framework has the potential to integrate ecological and genomic data. For this promise to be achieved however, the availability of metadata on the provenance and biogeochemical/ecological identification of the underlying biological samples is essential. Environmental metadata give essential context for genome data but current isolation of metadata resources (GOLD (Mukherjee et al., 2019) and NCBI’s BioSample (Barrett et al., 2012)) and lack of rich ontological and data standards hinder interoperability and reusability. Reusability of metadata is further hampered by inability to download metadata in bulk. Even within a single resource with a relatively consistent data schema, the fill rates for the existent terms are very low leading to existence of a large number of genomes without any usable metadata. For example, within 162,711 bacterial and archaeal GOLD genomes (accessed on 04/2021), only 17% had the Ecosystem field (GOLD: Study Fields: Ecosystem) completed with one of the three categories (Environmental, Engineered, or Host). Among the Environmental genomes, only ∼41% (7,868 genomes) had even the broadest ecosystem classification completed (GOLD: Study Fields: Ecosystem Category) leaving an overwhelming majority of genomes unusable. For a trait-based framework to fulfill its full potential in elucidating microbial trait-environment relationships, significant community efforts towards higher quality metadata standards and metadata enrichment such as that led by National Microbiome Data Collaborative (NMDC, https://microbiomedata.org/) towards higher quality metadata standards and metadata enrichment will be much needed.

## Methods

### Implementation

*microTrait* is implemented in R. Besides R-base functions, it depends on R packages dplyr, tidyr, tidyverse, readr (Wickham, 2019; Hadley et al., 2018; Wickham et al., 2019; Wickham and Henry, 2019) for efficient data access, manipulation and storage, doMC (Weston and Calaway 2015) to implement multicore functionality. *microTrait* is available from https://github.com/ukaraoz/microtrait.

### Construction of a Gene HMM Database of Protein Families (*microTrait-HMM*)

We constructed an HMM database that model gene loci underlying functional traits (called *microTrait-HMM*) based on archaeal and bacterial sequence diversity from 1) genomes of cultured organisms, 2) single cell genomes, 3) metagenome-assembled genomes, and 4) metagenomes from environmental, host associated and engineered microbiome samples. For each gene loci, a profile HMM was trained as follows. Seed protein sequences were collected from the non-redundant IMG/M database (img_core_v400) based on “EC Number”, “Gene Symbol”, and “IMG Term and Synonym” (Chen et al., 2019). Multiple sequences alignments (MSA) were generated from the seed sequences using MAFFT with an accuracy-oriented parameter set (--maxiterate 1,000 --localpair--anysymbol) (Katoh et al., 2005). Profile HMMs were built with HMMER/hmmbuild (Eddy 2008). We call the set of HMMs *microTrait-HMM* (Supplementary Table S2)*.* All seed sequences, MSAs, and profile HMMs are available at https://github.com/ukaraoz/microtrait-hmm.

### Estimation of Life History Traits (Minimal Doubling Time and Optimum Growth Temperature)

To estimate minimal doubling time from genome-wide codon usage bias, *microTrait* uses gRodon R package (Weissman et al., 2021) using multiple linear regression models trained on the dataset of maximal growth rates compiled by Vieira-Silva and Rocha (Vieira-Silva and Rocha 2010). Optimum growth temperature is estimated with the multiple linear regression models based on the same features of tRNA and 16S rRNA genes, ORFs and translated ORFs determined by Sauer and Wang (Sauer and Wang 2019), but reimplementing their python pipeline in R as part of the *microTrait* package itself to increase computational efficiency.

### Inference of Guilds

Ecological guilds were inferred from *microTrait* trait matrix with variance partitioning and clustering analysis. Trait values for “count traits” were normalized by genome size to express them as “per base-pair genomic investments”. The normalized trait matrix was used to calculate genome-to-genome distances using Wishart distance metric for mixed variable data (Wishart 2003) as implemented in R kmed package. Wishart distance is similar to the Gower distance (Gower 1971) for mixed variable data but applies a variance weight rather than a range for the numerical variables and uses a squared distance component. The resulting distance matrix was used to cluster genomes using hierarchical clustering with complete linkage. Next, we quantified variance in the genome to genome distances as a function of the number of defined guilds. We first cut the tree from hierarchical clustering into clusters ranging from 2 clusters to the total number of genomes in the dataset. Then, for each cut that corresponds to a given number of clusters, we quantified the variance in the distance matrix using cluster identity as a source of variation (using adonis2 in R vegan package) and plotted the resulting coefficient of determination (R^2^) as a function of the number of clusters. This allows the user the option to pick the number of guilds capturing a given level of trait variance across the dataset, and vice versa. Given a threshold for a trait variance or a number of guilds, we then assign each genome to a guild based on the corresponding tree cut from hierarchical clustering. Finally, we visualize the trait profiles for the defined guilds using trait positivity as a metric.

## Data Availability

The original contributions presented in the study are included in the article Supplementary Material, further inquiries can be directed to the corresponding author.

## Supplementary Material

Supplementary Figure S1 Performance of *microTrait*-HMMs with respect to cross-reference to KEGG orthologous families (KO). Each point corresponds to a gene-level HMM with the estimated sensitivity (true positive rate) and specificity (as false positive rate or 1-specificity) corresponding to the scoring threshold that maximizes F-score. The inset shows the cumulative distribution for the maximum F-scores.

Supplementary Figure S2
*microTrait* runtimes. Distribution of running times for isolate and metagenome-assembled genome sets normalized for genome size (measured as time (minutes) per Mb of sequence). Each point in the distribution corresponds to a genome. The normalized running times depend on the genome content, with more HMM hits requiring longer processing.

Supplementary Figure S3 Refinement of functional guilds using *microTrait*.

Supplementary Figure S4 Example *microTrait* trait matrix for soil isolate genomes as in Figure 5A, in high resolution.

Supplementary Table S1
*microTrait* hierarchy. Hierarchical mapping of genome-derived features into ecological function of increasing granularity in *microTrait*. *microTrait* hierarchy is an unbalanced hierarchy with 3 levels, with certain leaves spanning all 3 levels. References supporting the inference of traits from genome derived features are given in Supplementary Table S8.

Supplementary Table S2
*microTrait* HMMs. List of gene-level HMMs underlying *microTrait* pipeline (“*microTrait*-HMMs”), with cross-references (“dbxref”) to KEGG, EC, and Transporter Classification Database.

Supplementary Table S3 Evaluation of *microTrait* HMMs. Performance of *microTrait*-HMMs with respect to cross-reference to KEGG orthologous families (KO). For each model, the model score maximizing F-score for the corresponding KO is used as a trusted cutoff.

Supplementary Table S4
*microTrait* rules. Each *microTrait* rule is a boolean expression for presence/absence of *microTrait* HMMs or other *microTrait* rules.

Supplementary Table S5 Mapping of *microTrait* rules to the *microTrait* hierarchy. *microTrait* traits are either of type binary or count. Count traits can be counted by themselves or by their substrate (microtrait_rule-type = “count_by_substrate”) in case of transporters. Refer to ST6 for the mapping between substrates and the *microTrait* hierarchy.

Supplementary Table S6 Classification of substrates for substrate uptake and degradation by chemical class.

Supplementary Table S7
*microTrait* traits by strategy, type (i.e. binary, count), and granularity.

Supplementary Table S8 References for genome-derived features underlying ecological traits.

Supplementary Table S9 Selected GOLD genomes of organisms isolated from aquatic or terrestrial environments. Environmental isolate genomes (GOLD_organisms:Cultured == “Yes” AND GOLD_organisms:Ecosystem == “Environmental”) from GOLD database (https://gold.jgi.doe.gov/) were selected and filtered using ecosystem category and sample collection site (GOLD_organisms:Ecosystem Category == “Aquatic OR Terrestrial” OR GOLD_organisms:Sample Collection Site (MIGS-13) == “soil OR sediment OR rhizosphere”).

Supplementary Table S10 Taxonomic breakdown of selected GOLD genomes.

Supplementary Table S11 Mapping between taxa and functional groups based on Faprotax database. Faprotax (Functional Annotation of Prokaryotic Taxa) (http://www.loucalab.com/archive/FAPROTAX/lib/php/index.php?section=Download) is a database that maps prokaryotic clades (e.g. class, order, family, genus, species) to metabolic functions. For comparison with *microTrait* rules for the same metabolic functions, we resolved the listed taxa names to standard names, which are listed in this table (column: taxa).

Supplementary Table S12 Mapping of Faprotax taxa name to the NCBI taxa name.

Supplementary Table S13 Functional group assignments with Faprotax and *microTrait*. Each GOLD genome was assigned to a Faprotax functional group by taxonomy (i.e. based on Faprotax database as in ST11) and by *microTrait* (i.e based on genome sequence).

Supplementary Table S14 Evaluation of *microTrait* traits (genome-based) with respect to Faprotax functional groups (taxonomic name based). For each functional group, validity of *microTrait* predictions is evaluated based on Faprotax classifications (T: number of *microTrait* predicted positive genomes, N: number of *microTrait* predicted negative genomes, TP: number of true positive genomes, TN: number of true negative genomes, FP: number of false positive genomes, FN: number of false negative genomes, TPR: true positive rate, TNR: true negative rate).

Supplementary Table S15 Correlations between traits. Spearman’s rank correlation coefficient between pairs of traits.

Supplementary Table S16 Guild trait profile matrix. Trait profiles (*microTrait* granularity 3) for defined guilds as mean trait values.

Supplementary Table S17 Guild taxonomic profiles. Taxonomic profiles for defined guilds as relative abundance of genome taxonomy (phylum, class, order, family, genus).
