# Supplementary figures and images for: microTrait: A Toolset for a Trait-Based Representation of Microbial Genomes
Source: Front Bioinform. 2022 Jul 22;2:918853. doi: 10.3389/fbinf.2022.918853 (PMC9580909; doi:10.3389/fbinf.2022.918853)

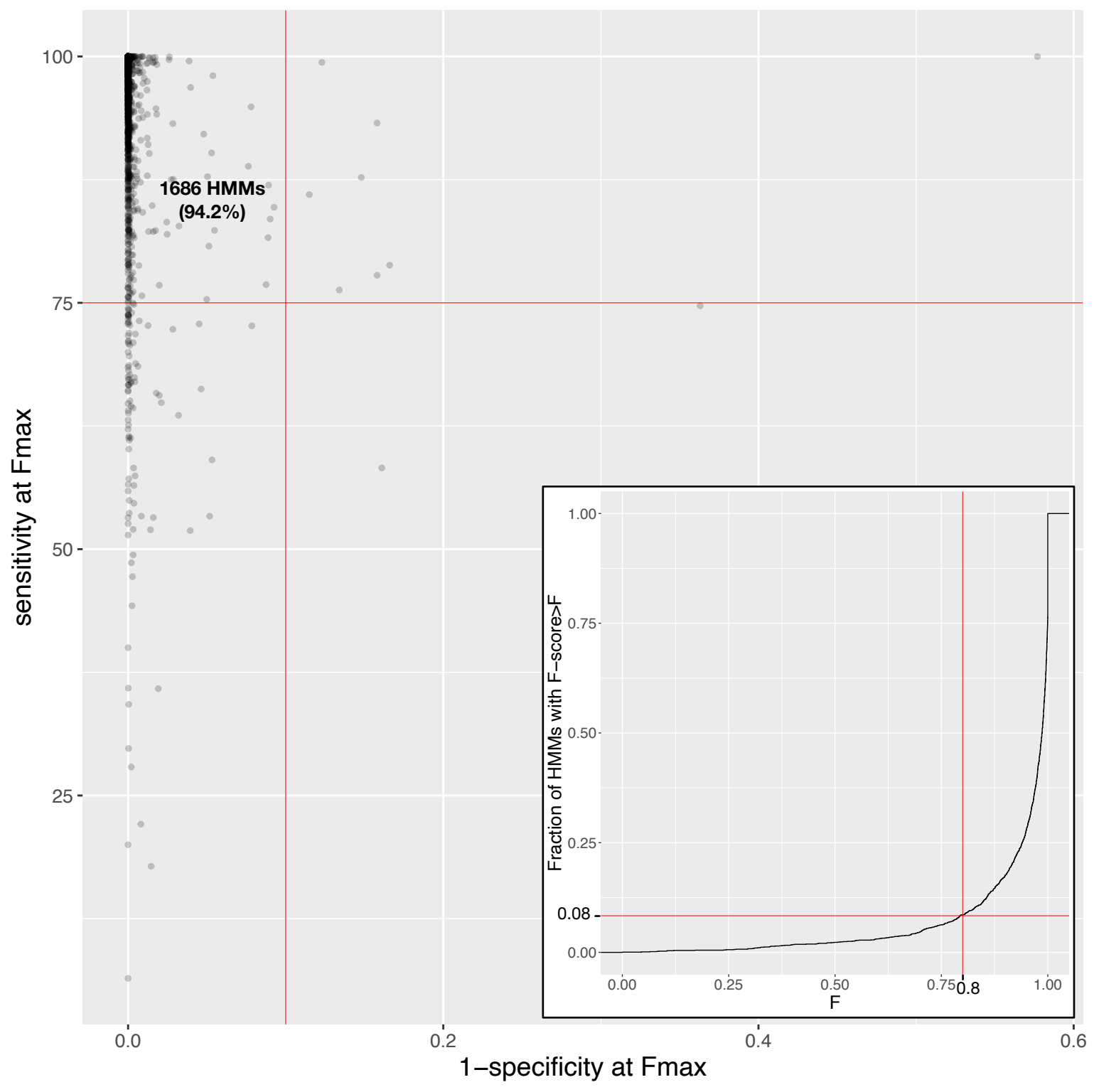

Supplement: Supplementary file 2 [file Image2.pdf]

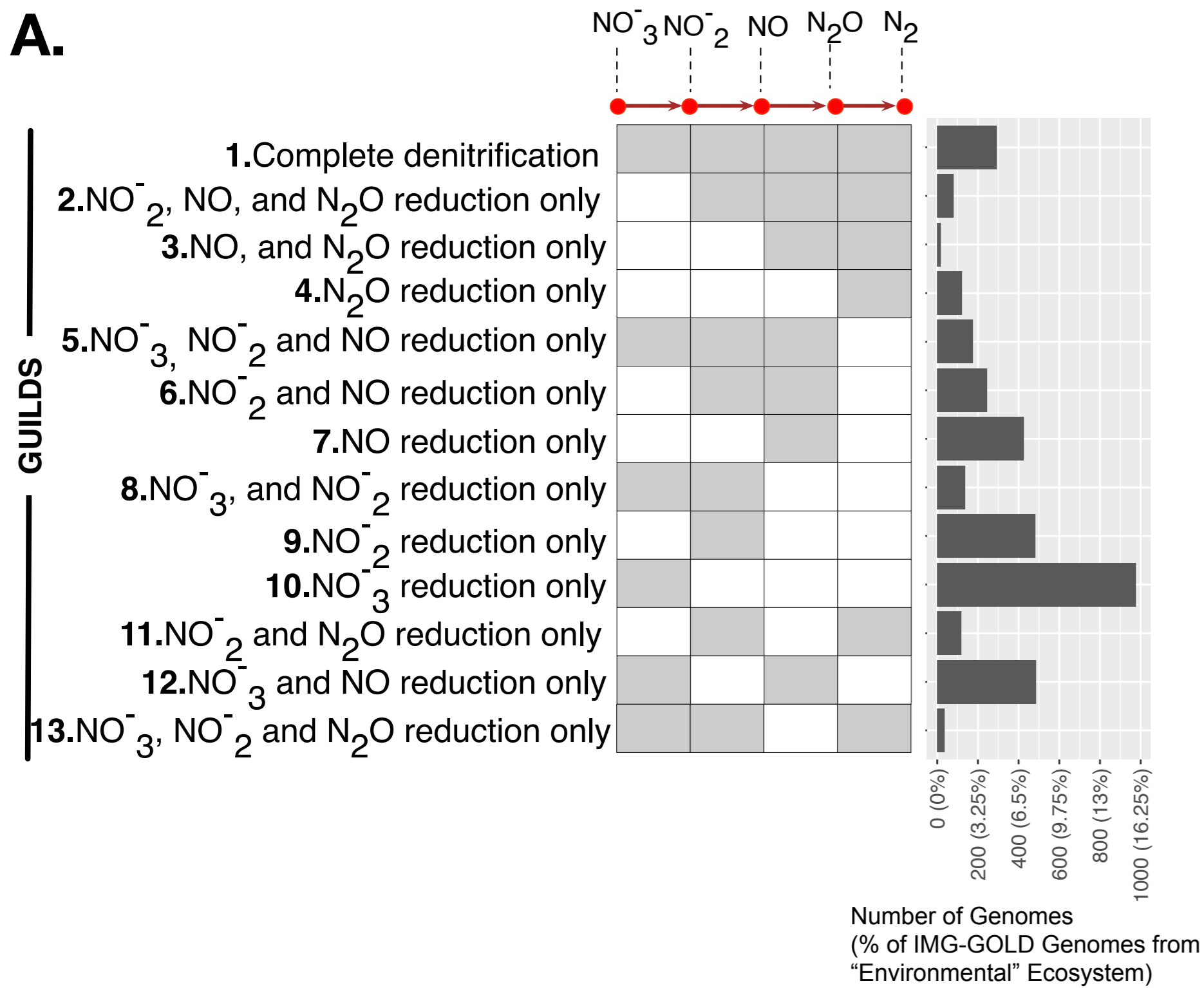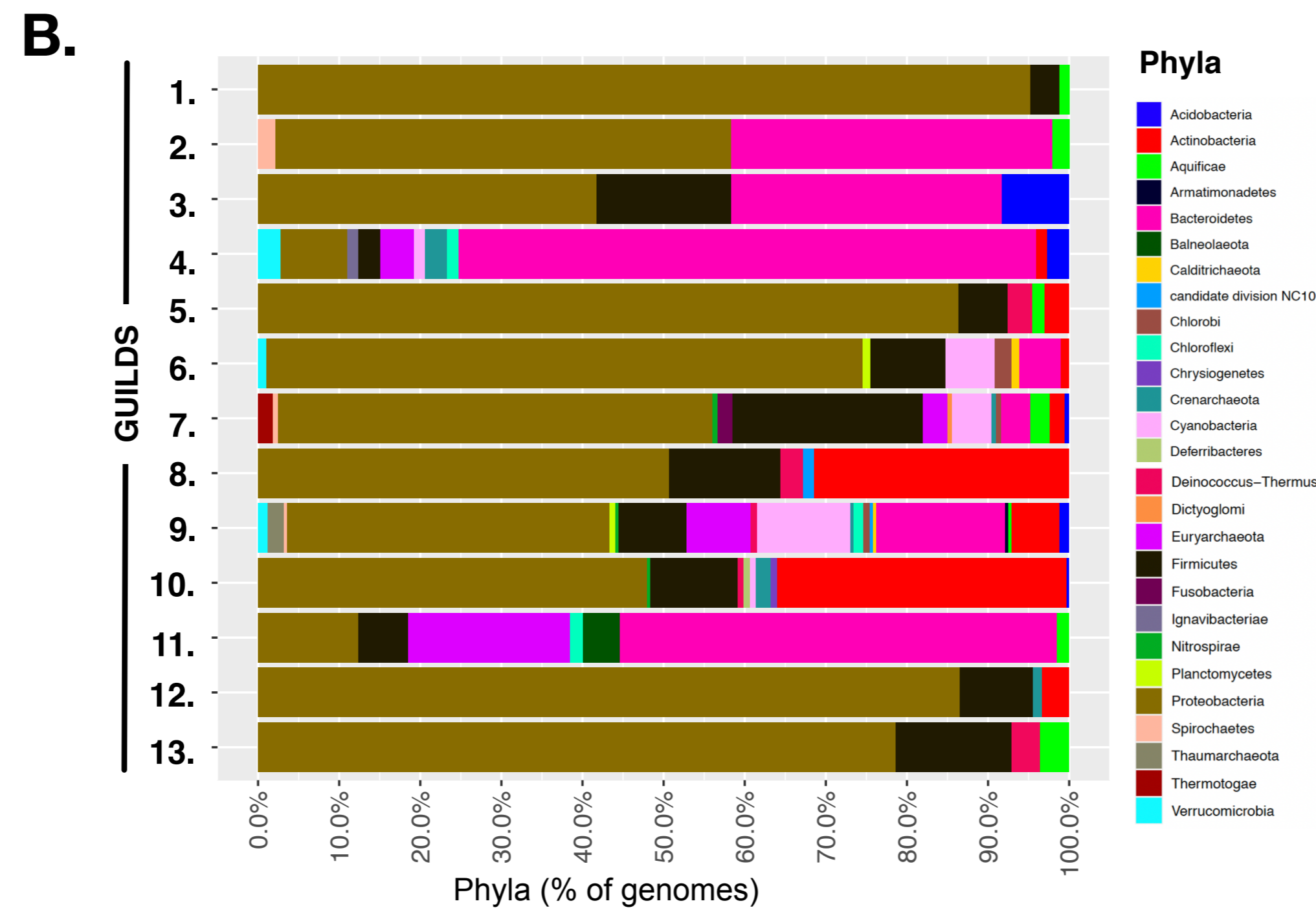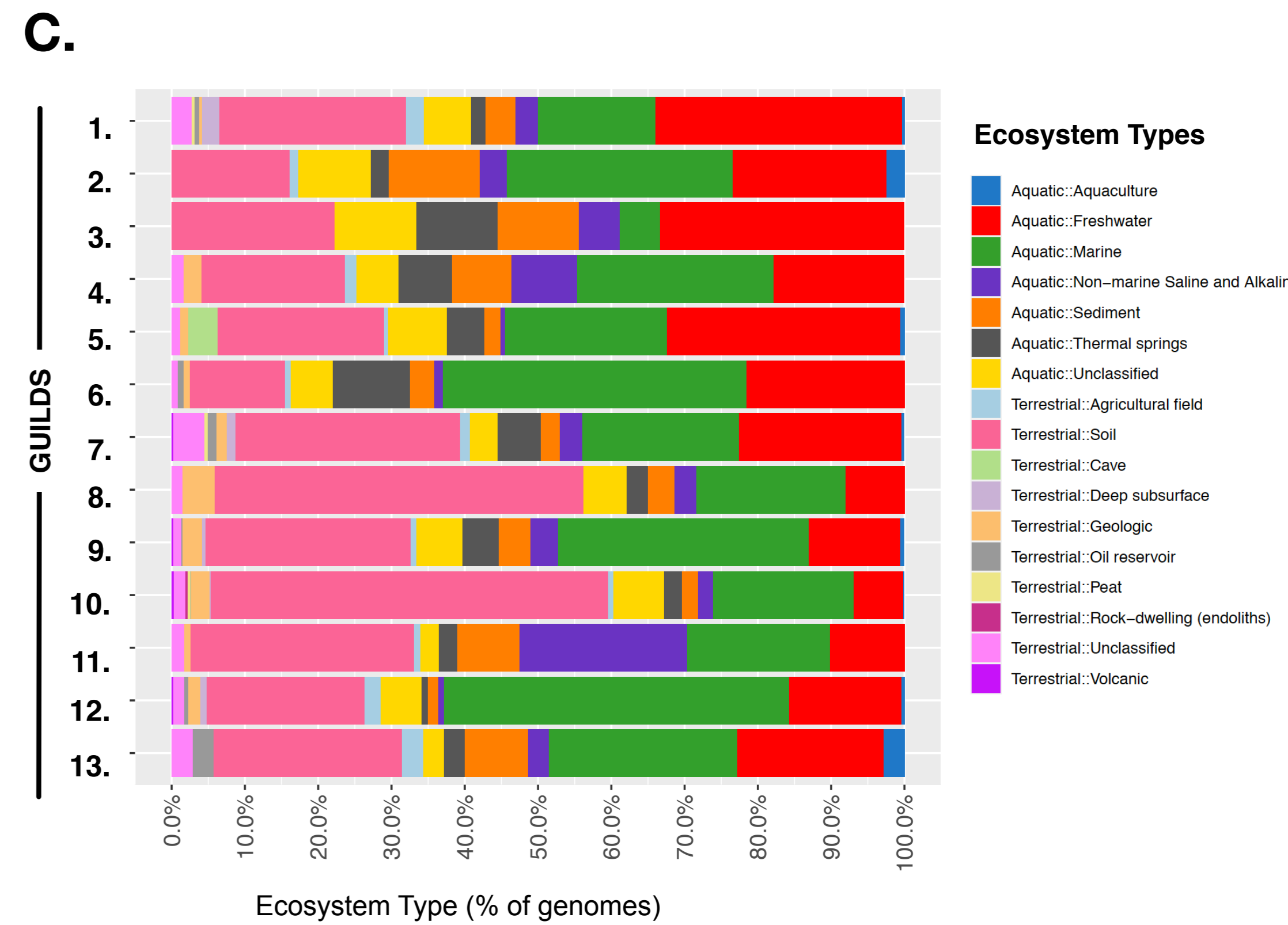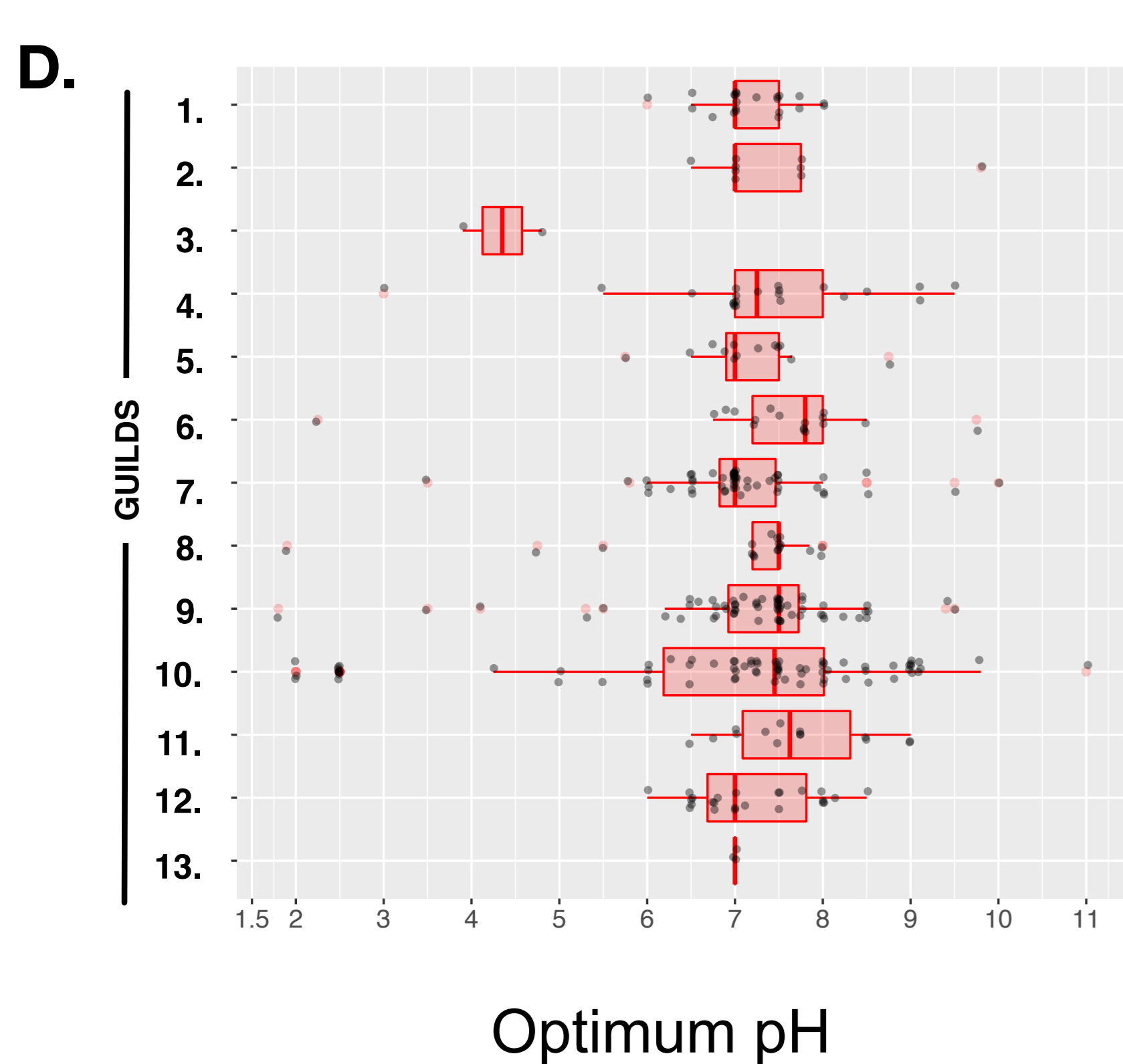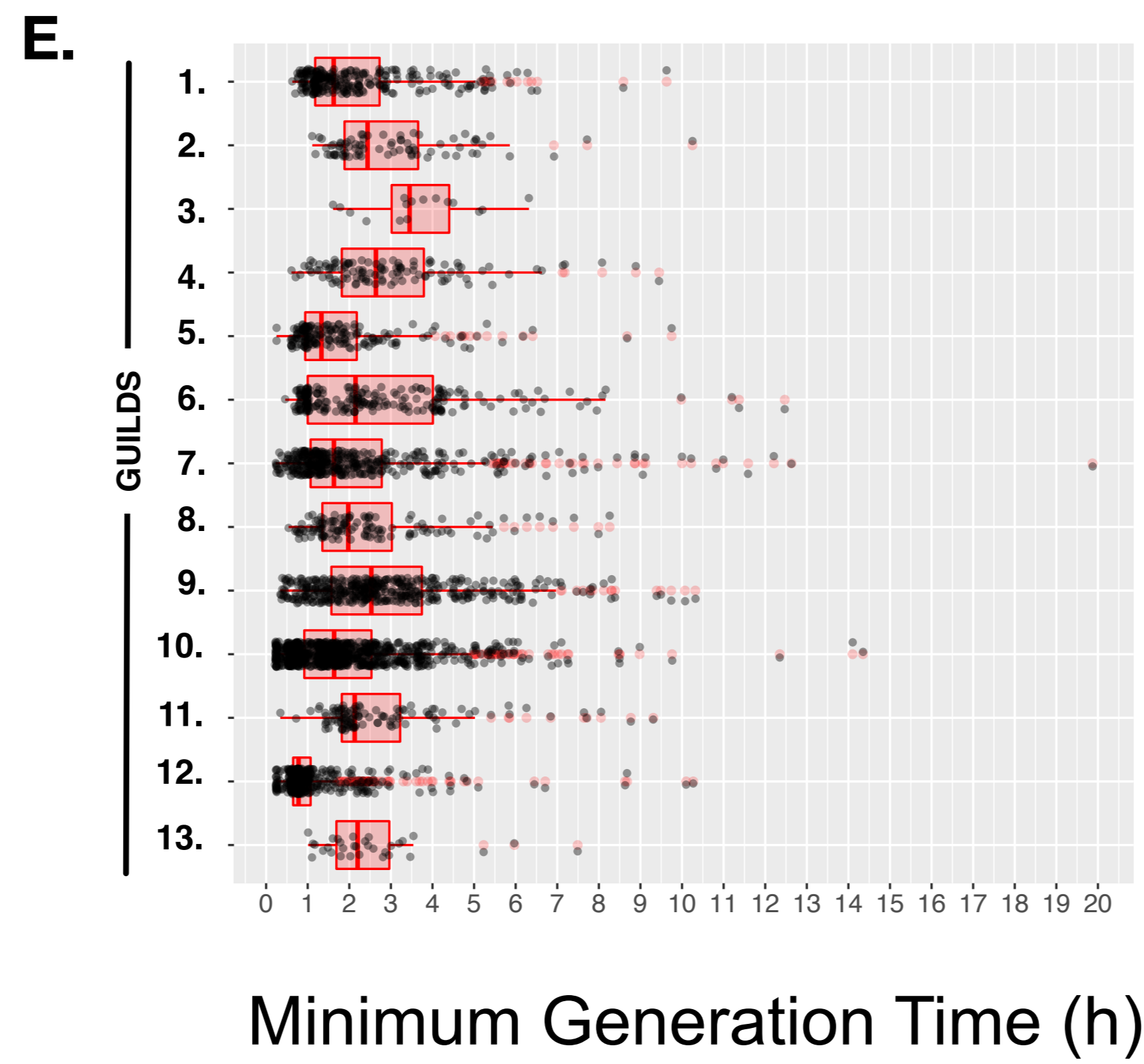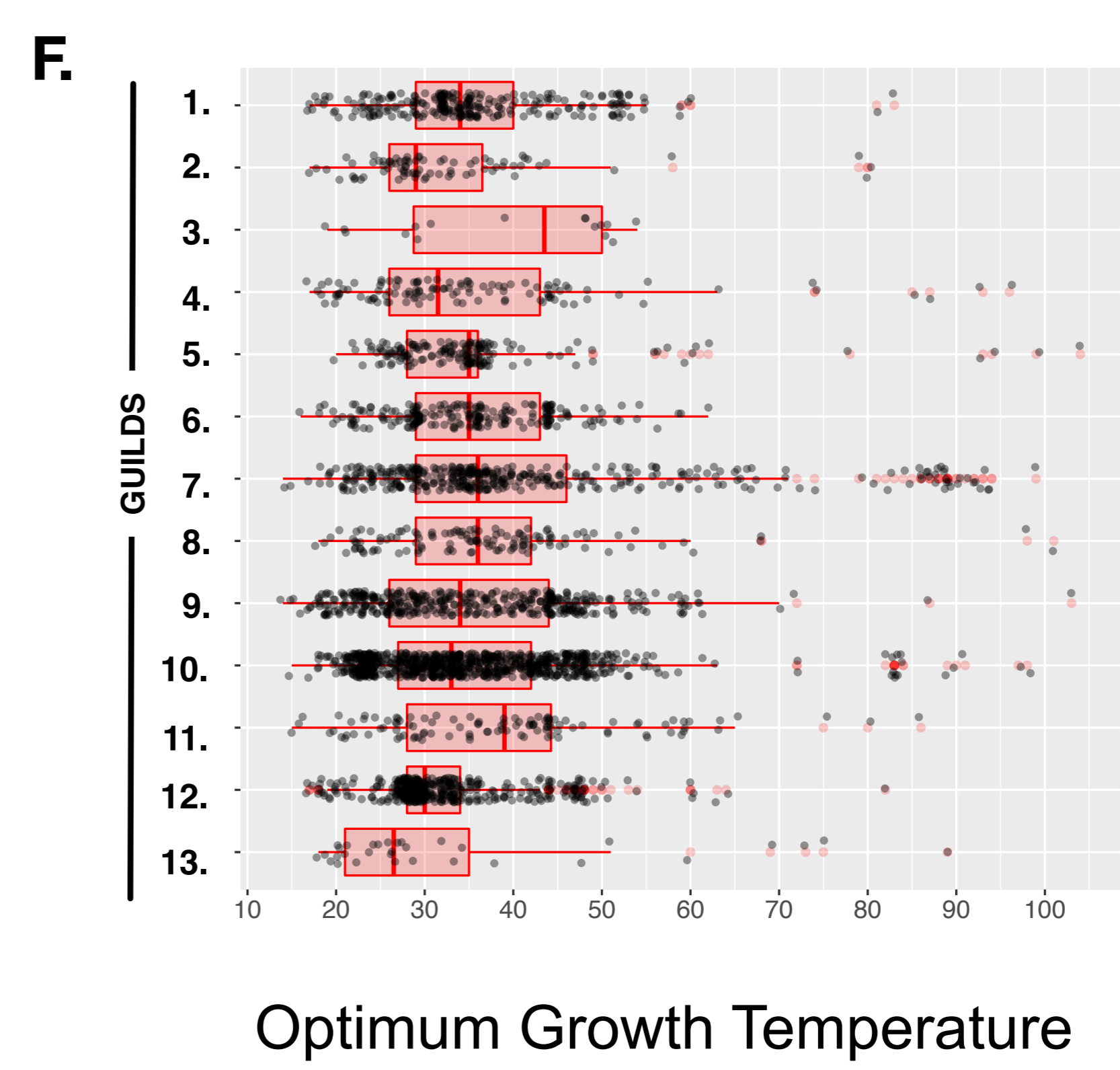

Supplement: Supplementary file 3 [file Image3.pdf]

dataset

PRJNA386568\_Woodcroft -

PRJNA362212\_Dombrowski -

PRJNA288027\_Anantharaman -

isolate -

0.5 1.0 2.0 3.0 4.0 5.0 6.0

minute/Mb

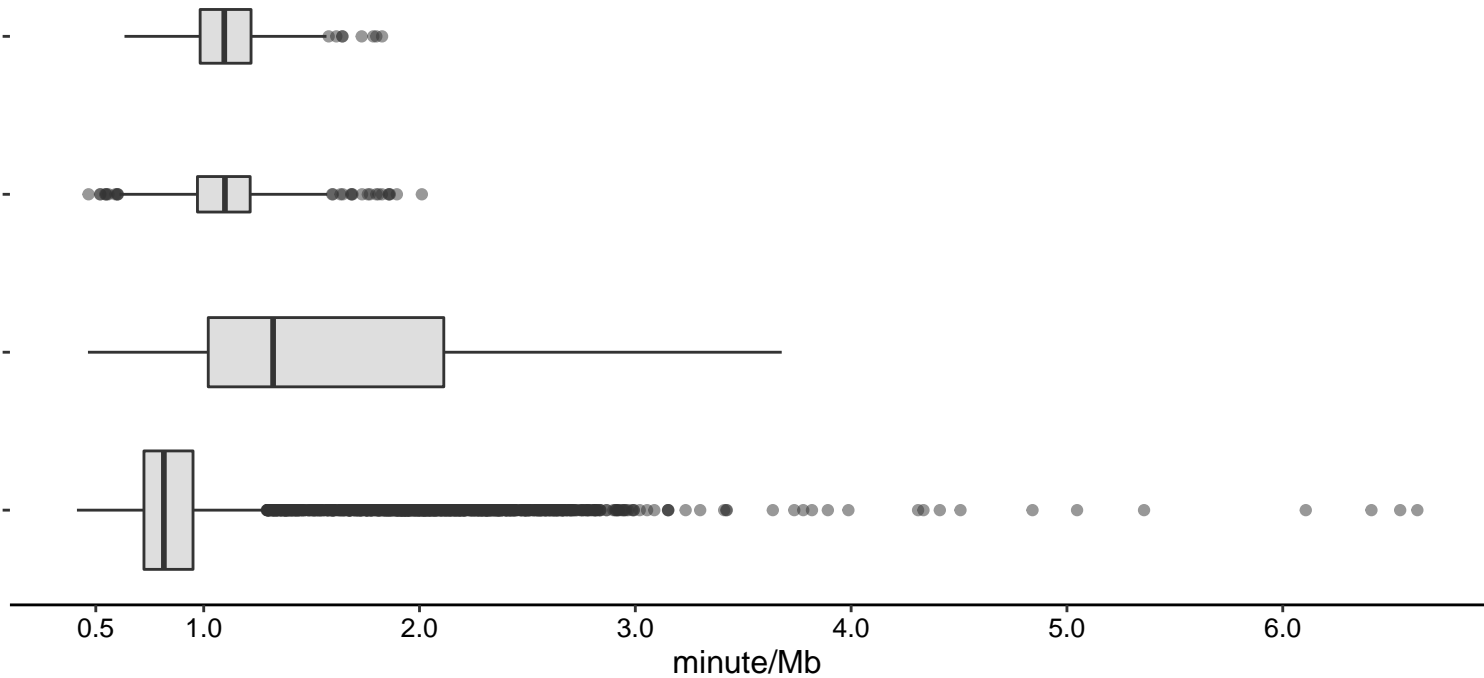

Supplement: Supplementary file 5 [file Image1.pdf]
