# Supplementary material for: microTrait: A Toolset for a Trait-Based Representation of Microbial Genomes
Source: Front Bioinform. 2022 Jul 22;2:918853. doi: 10.3389/fbinf.2022.918853 (PMC9580909; doi:10.3389/fbinf.2022.918853)
Supplement: Supplementary file 1 [file Image4.pdf]

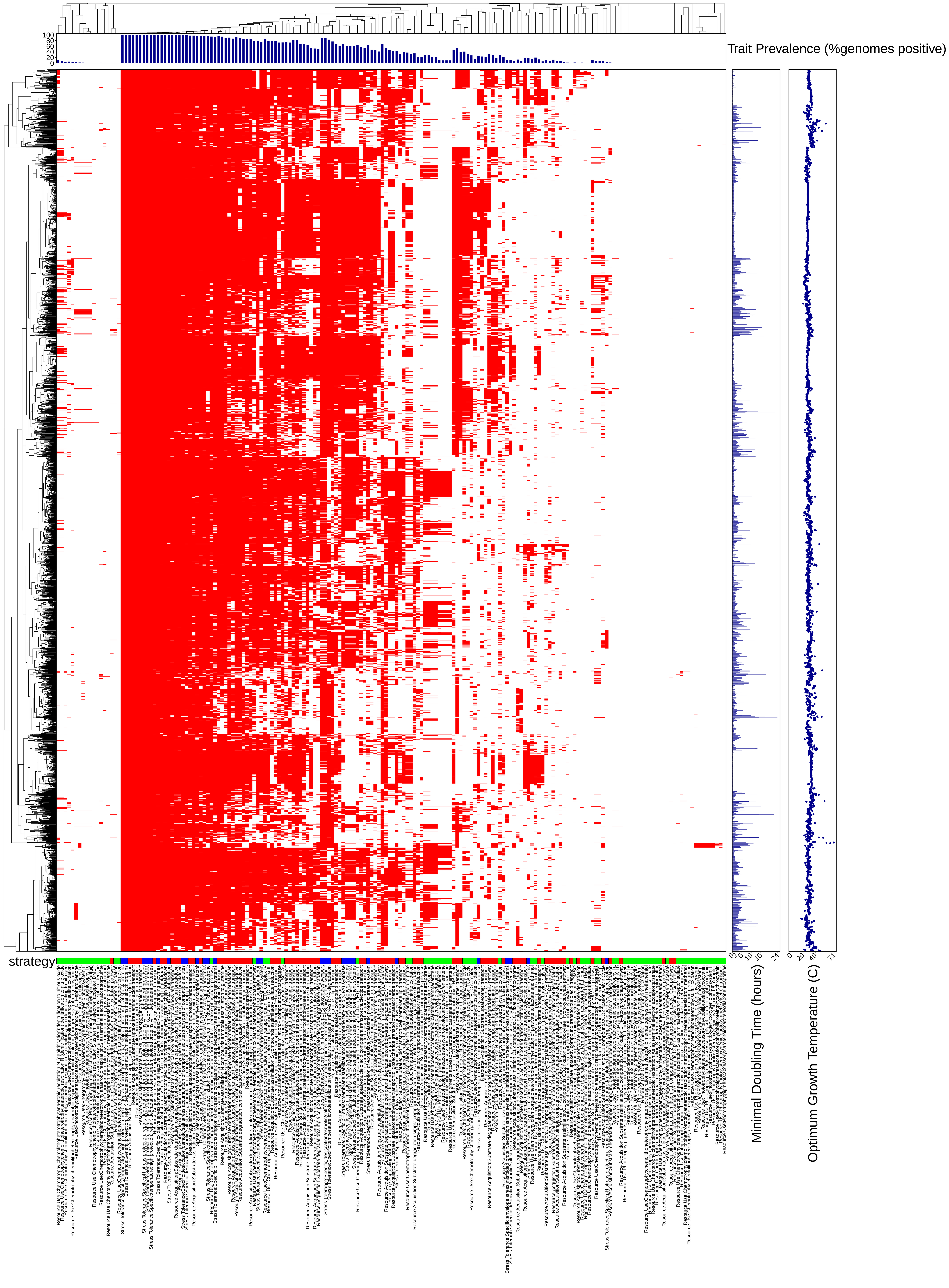
Trait Prevalence (%genomes positive)
100
80
60
40
20
0
strategy
Minimal Doubling Time (hours)
Optimum Growth Temperature (C)
